# The LL-100 Cell Lines Panel: Tool for Molecular Leukemia–Lymphoma Research

**DOI:** 10.3390/ijms21165800

**Published:** 2020-08-13

**Authors:** Hans G. Drexler, Hilmar Quentmeier

**Affiliations:** 1 Department of Human and Animal Cell Lines, Leibniz-Institute DSMZ-German Collection of Microorganisms and Cell Cultures, 38124 Braunschweig, Germany; hqu@dsmz.de; 2 Faculty of Life Sciences, Technical University of Braunschweig, 38124 Braunschweig, Germany

**Keywords:** cell lines, genetics, genomics, leukemia, lymphoma, model, panel

## Abstract

Certified cell line models provide ideal experimental platforms to answer countless scientific questions. The LL-100 panel is a cohort of cell lines that are broadly representative of all leukemia–lymphoma entities (including multiple myeloma and related diseases), rigorously authenticated and validated, and comprehensively annotated. The process of the assembly of the LL-100 panel was based on evidence and experience. To expand the genetic characterization across all LL-100 cell lines, we performed whole-exome sequencing and RNA sequencing. Here, we describe the conception of the panel and showcase some exemplary applications with a focus on cancer genomics. Due diligence was paid to exclude cross-contaminated and non-representative cell lines. As the LL-100 cell lines are so well characterized and readily available, the panel will be a valuable resource for identifying cell lines with mutations in cancer genes, providing superior model systems. The data also add to the current knowledge of the molecular pathogenesis of leukemia–lymphoma. Additional efforts to expand the breadth of available high-quality cell lines are clearly warranted.

## 1. Leukemia–Lymphoma Cell Lines Are Powerful Research Tools

The rationale for using leukemia–lymphoma (LL) cell lines is that they can provide exceptional opportunities to identify valuable information for a deeper understanding of cancer biology complemented by efforts in the development of new therapeutic approaches.

With many research projects becoming increasingly molecularly based, genomic characterization of LL cell lines is ever more essential. As sequencing costs have declined significantly, next generation sequencing (NGS) has been applied as a practical strategy for interrogating many genes simultaneously. Indeed, a number of LL cell lines have now been genomically sequenced with data also available on their gene expression profiles [1,2,3]. These early efforts at characterizing the genomic landscape were quite impressive and have led to the conjecture that systematic approaches would provide unprecedented amounts of pivotal data sets. Recent years have truly seen a welcome expansion of the scientific knowledge potential of LL cell lines [4]. Panels of specific LL cell line subtypes have also been extensively characterized [5,6,7,8] and presented as models for addressing unique topics, for example, to explore therapeutic innovations and to illuminate mechanisms of chromatin and transcriptional regulation in tumor cells [9].

Taking advantage of this significant technological momentum, we envisaged to close any knowledge deficits in the genomic landscape of a panel of the most valuable LL cell lines, covering the whole spectrum of leukemias and lymphomas (including here, myeloma). The manner in which this panel evolved from our long-time experience with these cell lines and some exemplary applications as tools for genetic discovery will be the focus of this article.

## 2. Major Advantages and Key Features of Leukemia–Lymphoma Cell Lines

The main reason for the use of LL cell lines is their ability to provide an unlimited source of cellular material that is able to grow indefinitely in vitro [10] (Table 1). Furthermore, cell lines can be stored in liquid nitrogen for an extended time (as long as the tank is refilled with liquid nitrogen) and can be recovered without any detrimental loss of cellular features or cell viability (if cells were frozen according to state of the art procedures). The absence of contaminating normal cells is also of importance. Finally, identical cell material can be made available to the worldwide scientific community [11].

Human leukemia–lymphoma cell lines are characterized by the six following general features (Table 1): (1) It is the common consensus that they originate from one cell and hence are of monoclonal origin. However, occasionally subclones may derive simultaneously from different original cells which will survive in vitro and co-exist even during long-term culture (more on this topic below). (2) Cellular differentiation is blocked at defined stages along the maturation axis. (3) The cultured cells proliferate continuously and autonomously, without the external addition of any growth factors; nevertheless, some specific cell lines were first developed in the late 1980s which were constitutively growth factor-dependent and required the addition of such supplements. (4) The features of cell lines are stable, including the cytogenetic and genetic characteristics—however, only under standard and optimal cell culture conditions. Once cells in a culture are selected due to culture pressure, subclones with divergent features will certainly emerge. All too often, the literature tends to spread the myth that “cell lines are inherently unstable”. It appears that in the majority of instances this alleged inherent instability is caused by improper and inadequate handling and culture of cell lines. For example, extended culture of LL cell lines will certainly lead to outgrowth of the “fittest cells” at the expense of slower growing or otherwise disadvantaged cells.

(5) LL cell lines contain genetic alterations: a survey of the LL cell line entries in a comprehensive compendium showed that among 637 well-characterized cell lines for which karyotypes have been published, only three (0.5%) showed a normal karyotype without any structural or numerical aberrations. However, in two of these latter cell lines extended cytogenetic analysis found both numerical and structural alterations [11].

Besides these gross alterations at the cytogenetic level, all cell lines also carry alterations which are detectable only at the molecular level, e.g., point mutations or deletions in a plethora of genes. These genetic changes presumably provide the affected cell with either proliferative or survival advantages and are thought to play an important role in both the in vivo tumorigenesis and the in vitro establishment of the cell line.

(6) Several studies have shown that leukemia–lymphoma cell lines retain gene expression patterns characteristic of tumor cells from patients with similar types of malignancy. An exception may be genes responsible for immortalization which are generally upregulated in tumor cell lines. There have been no longitudinal studies reported hitherto which document significant changes in gene expression from patient to cell line.

LL cell lines do have limitations as they were often derived from patients with end-stage disease which carry an extensive and selective mutational load. Thus, the initial cell cultures presumably contain heavily mutated cells, already optimized for in vitro growth [12]. In a literature survey of some 554 published LL cell lines, we found that 54% were established from patients at relapse or at a refractory/terminal disease stage, while 34% were derived from patient samples at diagnosis or at initial presentation [11]. Among the available ten bona fide Hodgkin lymphoma cell lines, 10/10 were derived from patients in terminal/refractory stages, 7/10 from pleural effusions (a sign of advanced disease), and 8/10 from nodular sclerosis-subtype Hodgkin lymphoma (at the expense of the three other histological subtypes) [7]. Hence, with regard to the successful establishment of new cell lines—at least in Hodgkin lymphoma—there is clearly a strong bias towards putatively more aggressive primary cells with specific and possibly more mutations.

The ability to grow autonomously in vitro may require transformation by a minimum of several oncogenic hits. This notion could help to explain why the repertoire of LL cell lines does not adequately represent each of the many clinical subentities and molecular subtypes [12]. For example for many fusion genes there exists no LL cell lines as models [11]. Assuming that investigators had also tried to establish cell lines from such cases, their failures (which commonly go unreported) confirm the generally very low success rate for establishing LL cell lines [11].

## 3. Intrinsic Differences between Primary Cells and Resulting Leukemia–Lymphoma Cell Line

Under the conditions of optimal cell culture, LL cell lines generally are stable and preserve the salient features of the original primary cells. Nevertheless, some have objected that the cells may take on certain properties during the attempts to establish a cell line and subsequently during continued culture as so-called in vitro artefacts. However, little evidence has been forwarded to substantiate such arguments. Alternatively, a specific feature of the neoplastic cells may facilitate immortalization or may serve as a prerequisite for immortalization.

This question whether certain cell line characteristics are obtained during in vitro growth or already exist in the original material should be addressed experimentally by directly comparing pairs of fresh neoplastic cells (at the single cell level) and the cell lines derived from them. While this issue may be seen as semantic, it nevertheless, is of importance as cell lines are widely used tools in experimental studies of an ever increasing panorama of scientific fields. For example, does the upregulation of critical tumor-associated genes truly reflect the molecular alteration which may cause the disease or its progression, resistance to therapy or other pathophysiological processes? If not, then obviously it would be legitimate to scrutinize the utility of cell lines as bona fide models for such investigations.

Finally, deliberate or unintended stress provoked by inappropriate cell culture conditions may lead to selection pressure. Intentional manipulations may result in phenotypic and genotypic shifts.

## 4. Continued Mutational Processes and Clonal Evolution in Leukemia–Lymphoma Cell Lines

Some have opined that LL cell lines carry an extensive and biased mutational repertoire, further optimized over years or even decades for in vitro growth [12]. 

Recently, it has been insinuated that selected cell lines (including a small panel of LL cell lines) constitute a substantial resource of live experimental and informative models for exploring questions related to mutational processes and their underlying mechanisms [13]. In some LL cell lines single cells were reported to continue to acquire mutational signatures over a prolonged cultivation time [13]. However, certain mutational signatures which were present in stock cell lines were clearly not generated during in vitro culture of their descendant clones, but had been observed in primary cancer cells [13,14]. Indeed, cancer cell lines show no evidence of genetic changes in major driver mutations over long-term in vitro cultivation and embody most of the spectrum of mutations that were detected in tumors, having similar patterns of chromosomal gains and losses as well as methylation regions [10]. Overall, it has been suggested that a certain mutation rate appears to be a common feature of in vitro culture [15].

Cancer cells frequently undergo genomic changes through proliferation, known as clonal evolution, resulting in intra-tumor heterogeneity [16]. This may also be observed in vitro in cell lines. However, clonal evolution of tumor cells in vitro is different from that in vivo, causing the replacement of cell populations during serial passage [17,18]. This process is provoked by the continuous subculture leading to dilutions of cells, which is certainly unavoidable during permanent cell culture over weeks, months and years (which we do not advise). Some cell lines have also been described that contain subclones that can be traced back to the patient, hence serving as isogeneic tools for the study of clonal evolution [19,20]. Epigenetic intra-cell line heterogeneity may also cooperate to shape the evolutionary course of cell line clones [21,22].

## 5. Assembly of the LL-100 Panel

Cancer cell line panels are a useful resource for a myriad of scientific questions. The US National Institute of Cancer 60 human tumor cell line anti-cancer drug screen (NCI-60) was developed in the late 1980s as a disease-oriented in vitro drug discovery tool intended to supplant the use of transplantable tumors in animal models in drug screening [23,24]. Ultimately this panel represents nine distinct tumor types [25]. Later, its role has changed to that of a service screen supporting the cancer research community [24]. Importantly, it has been shown that the NCI-60 panel retains certain disease etiology signatures [26,27]. Subsequently, Japanese investigators established a 45 cell line panel, covering various solid tumor types, in efforts to mine chemosensitivity data with bioinformatics [28].

**Figure 1:**
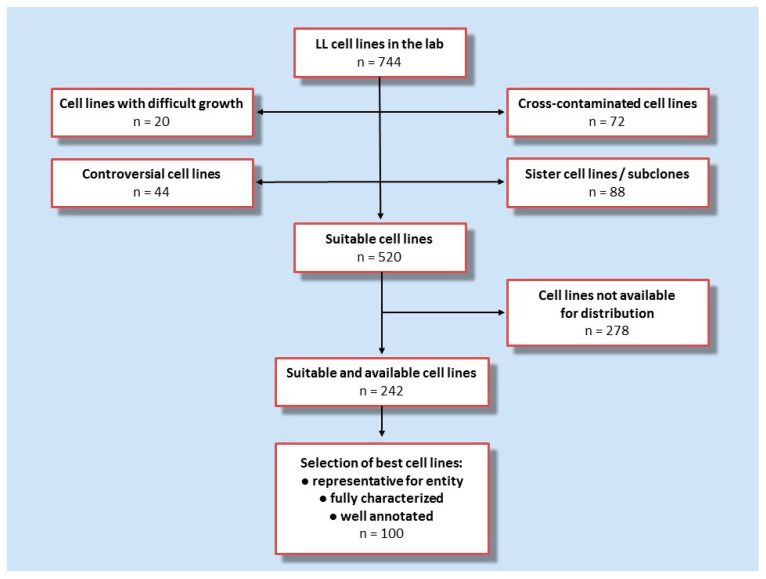
Flow chart showing the strategy for the assembly of LL-100 panel. Data on, and experience with, 744 cell lines in the lab were used to identify features which were considered exclusion criteria versus eligibility criteria. Exclusion criteria: difficult growth, cross-contamination, sister cell lines or subclones, or controversial cell lines including non-malignant Epstein–Barr-virus transformed B-lymphoblastoid cell lines—versus eligibility criteria: vigorous/robust proliferation, public availability, representative for a given entity, well-characterized and comprehensively annotated and classic/reference cell lines.

A few years ago, we already attempted to recommend sets of reference LL cell lines as the NCI-60 panel contains only five LL cell lines [29,30]. These initial efforts have reached maturity with recent work designing the current LL-100 panel. It is informative to review the approaches that we used to assemble the panel of 100 LL cell lines. More than 740 LL cell lines had been collected in the department. The application of negative and positive criteria led to the selection process as depicted in Figure 1. 

**Figure 2:**
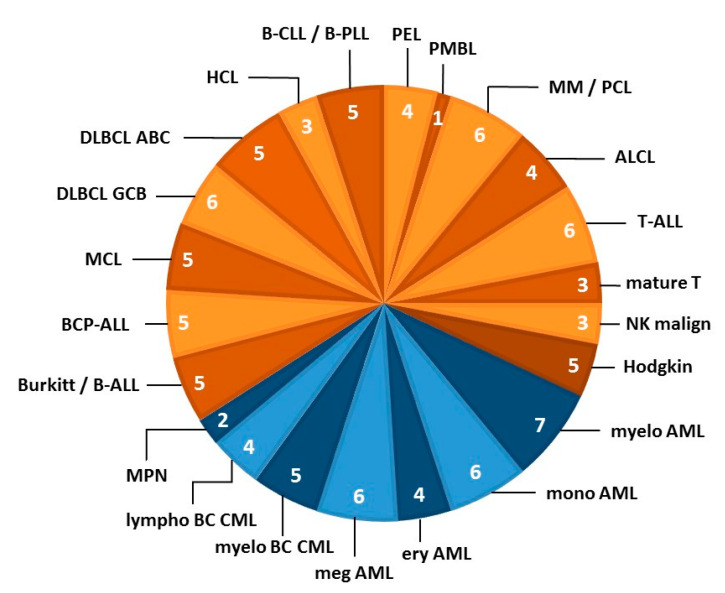
LL cell line stratification by disease category. The pie chart shows the 22 entities from which LL cell lines were derived that contributed to the LL-100 panel. Blue segments organized by entity represent myeloid malignancies and orange segments indicate lymphoid neoplasms (based on the revised WHO classifications of myeloid and lymphoid neoplasms; references [31,32]). The number of cell lines per entity is indicated in each segment. Each subset of LL cell lines was specifically tailored to represent this entity. The figure concept was further developed from Drexler et al. [33]. Abbreviations of disease entities: ABC, activated B-cell; ALCL, anaplastic large cell lymphoma; ALL, acute lymphoblastic leukemia; AML, acute myeloid leukemia; BC, blast crisis; BCP, B-cell precursor; CLL, chronic lymphocytic leukemia; CML, chronic myeloid leukemia; DLBCL, diffuse large B-cell lymphoma; ery, erythoid; GCB, germinal center B-cell; HCL, hairy cell leukemia; LL, lympbolastic lymphoma; lympho, lymphoid; malign, malignancy; MCL, mantle cell lymphoma; meg, megakaryocytic; myelo, myelocytic/myeloid; MM, multiple myeloma; mono, monocytic; MPN, myeloproliferative neoplasm; NK, natural killer; PCL, plasma cell leukemia; PEL, primary effusion lymphoma; PLL, prolymphocytic leukemia; PMBL, primary mediastinal B-cell lymphoma.

First, cell lines known to be cross-contaminated, sister cell lines and subclones, controversial cell lines (including cell lines which are rather Epstein–Barr transformed B-lymphoblastoid cell lines and not malignant cell lines), and cell lines known to be difficult to culture were excluded which left some 520 cell lines in the selection pool. Cell lines which are not available for general distribution were subtracted, thus, 242 cell lines remained. As a final step in the development of this initiative, we build on the data collected over decades in the cell line repository. For the final set we prioritized cell lines on the basis of availability, robust proliferation, and being well-characterized and comprehensively annotated. This disease-oriented concept relies on the fact that the cell lines are considered to be representative for the disease entity. LL cell lines is an umbrella term, encompassing a variety of entities and subentities. The LL entities for which cell lines were selected are based on the two Revised WHO classifications of myeloid and lymphoid neoplasms [31,32] (Figure 2). 

A more detailed breakdown of all disease entities and the assigned cell lines, various prominent cytogenetic and genomic mutations of these cell lines, together with unique distinctive features, is provided in Table 2. However, only selective key examples of the chromosomal and genomic landscape alterations are shown, therefore, this table is by no means inclusive of all available data. In a careful manner we genomically characterized these curated LL cell lines using whole-exome sequencing and RNA sequencing [34]. The resulting data are of high quality, fit for purpose and publicly available.

The current panel is unique in regards to its sample size (*n* = 100), completeness of cell line data and availability of other genetic information. It is particulary noteworthy to stress their detailed clinical annotation and their comprehensive profiling (encompassing morphology, immunophenotyping, cytogenetics, molecular analysis, cell culturing). The strengths of the panel include, furthermore, the intensive identity and quality control to which the cell lines have been subjected (domains like authentication and exclusion of cross-contamination; documentation of freedom from inadvertent mycoplasm and viral contamination; references [40,41,42,43]). Panel development was absolutely contingent upon the ability to exclude cross-contaminated and non-representative cell lines. Some authors had voiced the suspicion that several cell lines in the NCI-60 panel are not appropriate as model systems for the tumors [44,45]. By way of background, it must be recognized that the generally increased risk of cross-contamination is indeed borne out by the now proven inclusion of “false cell lines” in the NCI-60 panel [46], emphasizing the importance to identity control the entire cohort.

## 6. Exemplary Applications

**Figure 3:**
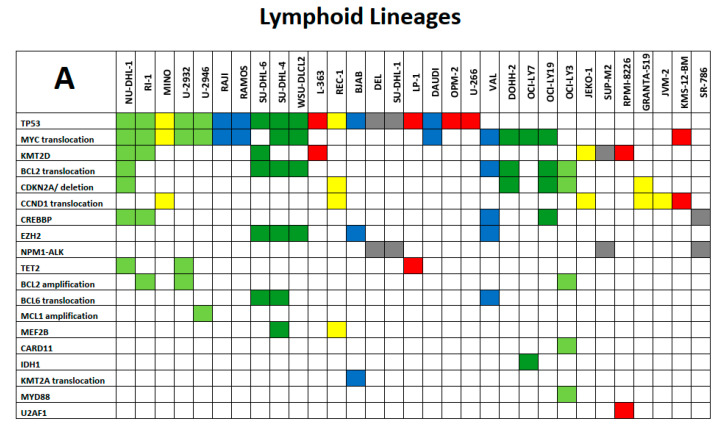

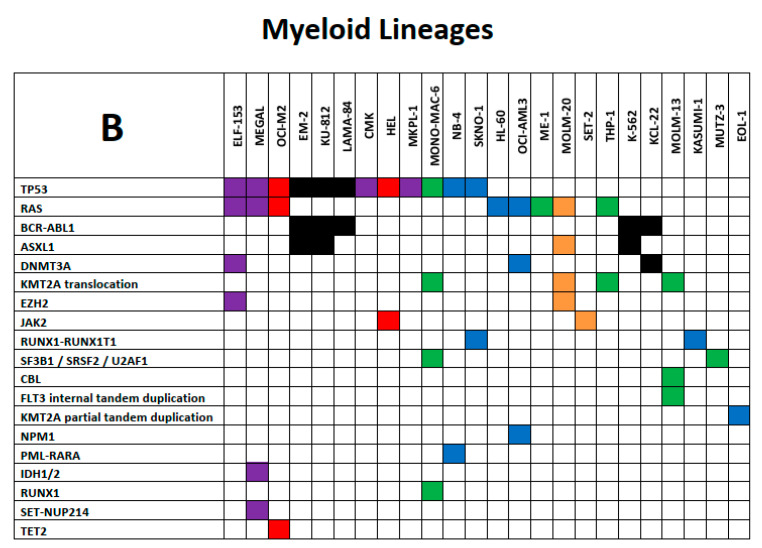
(**A**) and (**B**): Exemplary spectrum of selected mutational signatures in lymphoid and myeloid LL cell lines. Mosaic plot of mutant genes and chromosomal aberrations that are displayed in rows ordered by recurrence (top to bottom); cell lines are listed in columns. All mutations are annotated in the COSMIC database (hence carrying specific COSM numbers) and minimal allele frequency for mutation calling was set at < 0.01. (**A**) Color code of lymphoid LL cell lines: grey, ALCL; blue, Burkitt/B-ALL; light green, DLBCL ABC; dark green, DLBCL GCB; yellow, MCL; red, MM/PCL. (**B**) Color code of myeloid LL cell lines: blue, AML myelocytic; green, AML monocytic; red, AML erythroid; purple, AML megakaryocytic; black, CML myeloid blast crisis; orange, myeloproliferative neoplasms. The tables are not intended to be comprehensive across all aspects of leukemia–lymphoma-related alterations but instead to serve as focused high-priority areas.

We will limit our presentation of the utility of the LL-100 panel sequencing data on certain exemplary aspects. Selected mutational spectra of lymphoid and myeloid LL cell lines are shown as key examples in Figure 3A,B in the form of mosaic plots of mutant genes and chromosomal aberrations.

**Figure 4:**
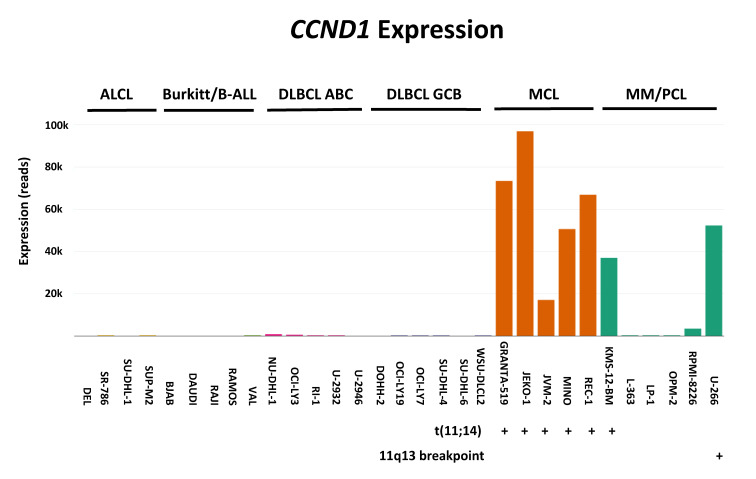
Example of gene overexpression. Here using RNA-sequencing we examined overexpression of *CCDN1* in cell lines derived from various lymphoma subgroups: anaplastic large cell lymphoma (ALCL); Burkitt lymphoma/B-acute lymphoblastic leukemia (B-ALL); diffuse large B-cell lymphoma (DLBCL) with its ABC (activated B-cell) and GCB (germinal center B-cell) variants; MCL; and MM/PCL. Note that 7/8 *CCND1*-positive cell lines carry aberrations affecting 11q13, the locus of the aberrantly expressed gene (five MCL and two MM/PCL cell lines).

Figure 4 gives examples of gene overexpression in the context of mantle cell lymphoma, attesting the fitness of these cell lines to reliably model this entity. Mantle cell lymphoma (MCL) is a distinct subtype of B-cell non-Hodgkin lymphoma which is characterized by the initiation driver event of t(11;14)(q13;q32) translocation leading to cyclin D1 upregulation (gene *CCND1*) and cell cycle dysregulation. The t(11;14)(q13;q32) is also one of the most common translocations in multiple myeloma (MM) and plasma cell leukemia (PCL).

**Figure 5:**
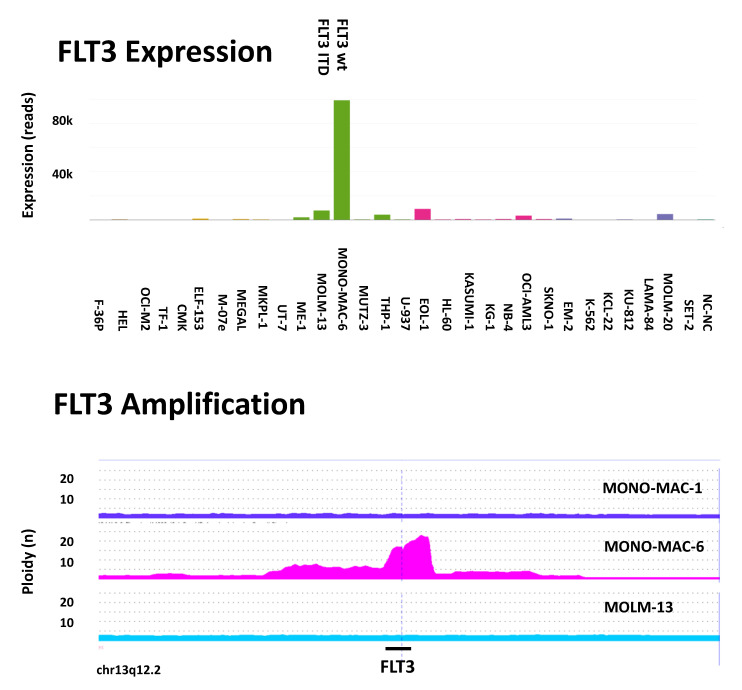
Example of gene overexpression and amplification. (Upper panel) RNAseq analysis revealed *FLT3* overexpression in cell line MONO-MAC-6 which is the wild-type (wt) in the FLT3 ITD analysis, whereas cell line MOLM-13 carries the *FLT3* ITD. (Lower panel) According to CGH (comparative genomic hybridization) array analysis, the chromosomal region of *FLT3* (13q12.2) is highly amplified in MONO-MAC-6 but is not amplified in sister cell line MONO-MAC-1 nor in MOLM-13. Hence, MOLM-13 and MONO-MAC-6 are *FLT3*-mutant cell lines whereas MONO-MAC-1 does not have an apparent *FLT3* mutation.

Another example is the gene *FLT3* (Figure 5). Fms-like tyrosine kinase-3 (*FLT3*) is a gene that encodes for a tyrosine kinase that is essential in the proliferation and differentiation of hematopoietic cells. *FLT3* is the most commonly mutated gene in AML. Mutations in *FLT3* most often occur as internal tandem duplication (ITD) and less commonly as point mutations, followed by gene amplification. 

These illustrative examples demonstrate that the LL-100 panel can provide insights into the relevance and validity of using cell lines as models for molecular and cellular research. 

## 7. Advantages and Benefits of the LL-100 Panel

The LL-100 panel offers several unique features, advantages and benefits (Table 3). All cell lines come from a single source (which is a public non-profit cell line repository) and are stringently validated, undergoing rigorous and continuous identity control for authentication and quality control for the documentation of freedom from mycoplasma and non-inherent viral contamination. Further immunological, cytogenetic, molecular biological, morphological and functional characterizations confirm derivation from and assignment to the presumed cell lineage and tissue. In order to avoid genetic drift during long-term culture and the emergence of subclones, cell lines are not in continuous culture but remain in frozen storage to keep the passage numbers as low as possible. Furthermore, the methods of RNA and DNA isolation, and sequencing in this endeavour are identical for all cell lines. Therefore, these datasets allow for comparative studies without methodical impact [34]. Finally, the generated data (whole exome sequencing and RNA seq) are at the free disposal of the scientific community; the cell lines are publicly available from the cell lines bank. A number of well-known LL cell lines which are used all over the world are included in the panel to provide a reference standard.

## 8. Conclusions and Future Directions

This historical perspective is intended to show the conceptual development and implementation of the LL-100 panel and its value and legitimacy as a research tool. Owing to the wide variety of different types of leukemias and lymphomas, the selection of a limited number of cell lines required choices and compromises to be made and entails various limitations.

However, our long-term accumulation of pertinent data and our years of experience with the cell lines has permitted the judicious selection of an adequate number of representative cell lines. Hence, we had the unique opportunity to collate in this initiative a panel of 100 cell lines that reflects the diversity of leukemia–lymphoma, stratified into 22 entities.

This panel draws its strength from a large size and the quality of the included cell lines. Unlike previous efforts at establishing cell line panels, from the beginning this initiative fully exploited modern karyotyping by the systematic application of classical and molecular cytogenetics, hence, there exists a deep characterization at the genetic level. 

The unchecked dissemination and reckless use of false LL cell lines is a serious drawback [47,48,49,50]. The assembly of a cell line panel is not only susceptible to selection bias but first and foremost to inclusion of cross-contaminated cell lines. However, in our endeavour, the latter constraint has been mitigated by an absolutely rigorous and structured identity control process of each cell line.

In order to strengthen the utility of the data for the scientific community, it is critical that the NGS data are freely and publicly available and that also the accompanying cell lines are readily available. These two requirements are here satisfactorily fulfilled.

Thus the LL-100 panel cell lines may help to take the understanding of leukemia–lymphoma biology to the next level, expanding the utility of cell lines and increasing their precision as experimental models for many applications [27]. For example, the availability of the comprehensive LL-100 panel should provide the framework to drive the molecular–genetic discovery of targetable alterations, thus providing additional scientific benefit.

Further existing and also novel LL cell lines should continue to be integrated in the systematic application of NGS, on one hand adding substantial depth to their obligatory characterization moving beyond traditional features of old and new cell lines, and on the other hand thus expanding the repertoire of informative cell lines. Clearly, to fully sequence a vast repertoire of the most important LL cell lines would be an ambitious, yet feasible, approach to move the field forward. There are valuable online tools that enable access to genomic and phenotypic datasets that were derived from cancer cell lines, including some LL cell lines [1,4,10,51,52,53,54]. Again, the potential of a significant therapeutic translational impact is contingent on the public availability of cell lines and their data. To advance, it will also be essential to portray comprehensively the whole spectrum of LL cell lines in order to capture the vast diversity that was observed in the patients [10].

In summary, the wealth of genomic data generated from cell lines will hopefully lead to an increase in the number of testable and actionable hypotheses in leukemia–lymphoma pathobiology, as envisaged previously [55]. In some selected examples, we were able to demonstrate sequence-level evidence of its successful application and robustness. Scientific discovery using LL cell lines has evolved considerably in the last few years and decades and it is reasonable to predict that LL cell lines are poised to contribute to significant innovations in the years to come. As leukemias and lymphomas are rare diseases, there is only limited access to patient samples and a reduced amount of available in vitro models. Therefore, it is essential to establish new human LL models that will provide enough biological material to perform functional and molecular studies [56]. The development of new, preclinical models of leukemia–lymphoma (including LL cell lines) that can capture the considerable genetic diversity has been recognized as a priority area for future research [12,57]. There is clearly a need for “high-quality” LL cell lines which are authenticated and fully annotated in all possible ways.

The key sequencing data have been deposited at the European Nucleotide Archive (ENA) under the accession number PRJEB30297 for WES and PRJEB30312 for RNA-seq, respectively.

## Figures and Tables

**Table 1:** Advantages and Key Features of Leukemia–Lymphoma Cell Lines.

**Major Advantages**
Unlimited supply of cell material
Worldwide availability of identical cell material
Infinite storability in liquid nitrogen and recoverability
Absence of contaminating normal material
**Key Features**
Monoclonal origin
Differentiation arrest at a discrete maturation stage
Sustained proliferation in culture
Stability of most features in long-term cultures
Specific genetic alterations
Retention of tumor gene expression patterns

Modified from Reference [11].

**Table 2:** The LL-100 Panel: Selected Key Genetic Features and Unique Characteristics.

Entity ^1^	Cell Line ^2^	Clinical Data (Diagnosis, Age, Disease Status) ^3^	Cytogenetics ^4^	Genomic Landscape ^5^	Unique Distinction ^6^
BCP-ALL	697 KOPN-8 NALM-6 REH SEM	pre B-ALL, child, at relapse pre B-ALL, infant, n.r. pre B-ALL, adolescent, at relapse pre B-ALL, adolescent, at relapse pre B-ALL, child, at relapse	t(1;9)(q23;p13) t(11;19)(q23;p13) t(5;12)(q33;p13) t(4;12;21;16)(q32;p13;q22;q24) t(4;11)(q11;q24)	*TCF3-PBX1* *KMT2A-MLLT1* *MSH2* del *ETV6-RUNX1, RUNX1-PRDM7* *KMT2A-AFF1*	reference cell line classic/reference cell line classic/reference cell line
B-NHL: Burkitt/B-ALL	BJAB DAUDI RAJI RAMOS VAL	African Burkitt, child, terminal African Burkitt, adolescent, n.r. African Burkitt, child, n.r. American Burkitt, child, n.r. B-ALL, adult, n.r.	t(11;17)(q23;q23) t(8;14)(q24;q32) t(8;14)(q24;q32) t(8;14)(q24;q32) t(8;14;18)(q24;q32)	*KMT2A-CLTC* *IGH-MYC* *IGH-MYC* *IGH-MYC* *MYC-IGH-BCL2, * r	EBV−, classic cell line EBV+, reference cell line EBV+, reference cell line triple-hit B-NHL cell line
B-NHL: CLL/PLL	HG-3 JVM-3 JVM-13 MEC-1 PGA-1	B-CLL, adult, at diagnosis B-PLL, adult, at diagnosis B-PLL, adult, at diagnosis B-CLL, adult, at diagnosis B-CLL, adult, at diagnosis	del(13)(q12q32) trisomy 12, del(13)	*R3HCC1L-HTRA1*	EBV+ EBV+ EBV+ EBV+ EBV+
B-NHL: DLBCL ABC	NU-DHL-1 OCI-LY3 RI-1 U-2932 U-2946	DLBCL, adult, n.r. DLBCL, adult, at relapse B-NHL, adult, terminal DLBCL, young adult, terminal DLBCL, adult, terminal	t(3;8)(p25;q24), t(14;18) t(14;19)(q32;q13) t(4;8)(q22;q24) t(8;14)(q24;q32) t(8;14)(q24;q32)	*IGH-BCL2*, *MYC* r *IGH-SPIB, MYD88* mut *BCL2* amp, *MYC* r *BCL2* amp *IGH-MYC*	double-hit B-NHL cell line double-hit B-NHL cell line 2 distinct subclones
B-NHL: DLBCL GCB	DOHH-2 OCI-LY7 OCI-LY19 SU-DHL-4 SU-DHL-6 WSU-DLCL2	B-NHL, adult, refractory DLBCL, adult, at relapse DLBCL, young adult, at relapse DLBCL, adult, n.r. DLBCL, adult, n.r. DLBCL, adult, at relapse	t(8;14;18)(q24;q32;q21) t(8;14)(q24;q32) r(8), t(14;18)(q32;q21) t(14;18)(q32;q21) t(1;3)(q42;q21), t(14;18)(q32;q21) t(3;8)(q27;q24), t(14;18)(q32;q21)	*IGH-MYC, IGH-BCL2* *IGH-MYC* *IGH-BCL2*, *MYC* r *IGH-BCL2*, *BCL6* r, *MYC* r, *EZH2* mut *IGH-BCL2*, *BCL6* r, *EZH2* mut *IGH-BCL2*, *MYC* r, *EZH2* mut	double-hit B-NHL cell line double-hit B-NHL cell line triple-hit B-NHL cell line double-hit B-NHL cell line triple-hit B-NHL cell line
B-NHL: HCL	BONNA-12 HAIR-M HC-1	HCL, adult, at diagnosis HCL, adult, terminal HCL, adult, at diagnosis		*IGH-TCL1A*	EBV+ EBV+ EBV+
B-NHL: MCL	GRANTA-519 JEKO-1 JVM-2 MINO REC-1	MCL, adult, refractory MCL, adult, leukemic conversion MCL, adult, at diagnosis MCL, adult, terminal MCL, adult, terminal	t(11;14)(q13;q32) t(11;14)(q13;q32) t(11;14)(q13;q32) t(11;14)(q13;q32) t(11;14)(q13;q32)	*IGH-CCND1* *IGH-CCND1* *IGH-CCND1* *IGH-CCND1* *IGH-CCND1*	EBV+, reference cell line EBV+, reference cell line
B-NHL: PEL	BC-3 BCBL-1 CRO-AP2 CRO-AP5	Non-AIDS PEL, adult, at diagnosis AIDS PEL, adult, n.r. AIDS PEL, adult, at diagnosis AIDS PEL, adult, terminal		*BCL6* mut *BCL6* mut, *MYC* amp *BCL6* mut *BCL6* mut	EBV− HHV8+ EBV− HHV8+ HIV− EBV+ HHV8+ HIV− EBV+ HHV8+ HIV−
B-NHL: PMBL	U-2940	DLBCL, adolescent, terminal		biallelic *SOCS1* del	
Multiple Myeloma / PCL	KMS-12-BM L-363 LP-1 OPM-2 RPMI-8226 U-266	Myeloma, adult, terminal PCL, adult, n.r. Myeloma, adult, terminal Myeloma, adult, terminal Myeloma, adult, at diagnosis Myeloma, adult, terminal	t(11;14)(q13;q32) t(4;14)(p16;q32) t(4;14)(p16;q32)	*IGH-CCND1, IGH-MYC* *NRAS* mut, *TP53* mut *IGH-NSD2* *IGH-NSD2* *MYC* ins, biallelic *TRAF3* del *PTEN* del	classic/reference cell line reference cell line classic/reference cell line classic/reference cell line
Hodgkin Lymphoma	HDLM-2 KM-H2 L-428 L-1236 SUP-HD1	Nodular sclerosis, adult, stage IV Mixed cellularity, adult, stage IV Nodular sclerosis, adult, stage IV Mixed cellularity, adult, stage IV Nodular sclerosis, adult, stage IV		*CCND2, JAK2* amp, *SOCS1* del *CIITA-C15ORF65*, *BCL6* mut, *JAK2* *BCL6* mut, *EZH2* mut, *JAK2* amp biallelic *SOCS1* mut, *STAT6* amp	reference cell line reference cell line reference cell line
T-ALL / T-Lymphoblastic lymphoma	CCRF-CEM DND-41 HPB-ALL JURKAT MOLT-4 RPMI-8402	ALL, child, terminal ALL, child, n.r. T-ALL, child, at diagnosis ALL, child, at relapse ALL, adolescent, at relapse ALL, adolescent, n.r.	t(5;14)(q35;q32) t(5;14)(q35;q32) del(1)(p32), t(11;14)(p15;q11)	*NKX2.5-BCL11B* *TLX3-BCL11B* *CBFB-MYLPF, TLX3-BCL11B* *FBXW7* mut, *PTEN* del, *TP53* mut *CDKN2A* del, *NRAS* mut *LMO1-TRD, STIL-TAL1*	classic/reference cell line classic/reference cell line classic/reference cell line classic/reference cell line
Mature T-malignancy	DERL-7 HH MOTN-1	T-NHL, adult, at progression CTCL, adult, terminal T-LGL, adult, chronic phase	t(7;16)(q11;p13)	*CDKN2A* del, *SLFN13* del *FOXK2-TP63* *CASP8-ERBB4, TBL1XR1-TP63*	rare hepatosplenic T-cell line rare T-LGL cell line
NK malignancy	KHYG-1 NK-92 YT	NK leukemia, adult, at diagnosis LGL-NHL, adult, at diagnosis ALL, adolescent, at relapse		*BCL2* cna, *MYC* cna	EBV+, reference cell line EBV+, reference cell line
ALCL	DEL SR-786 SU-DHL-1 SUP-M2	Mal. histiocytosis, child, at diagnosis NHL, child, n.r. Mal. histiocytosis, child, at diagnosis Mal. histiocytosis, child, refractory	t(2;5;6)(p23;q35;p21) t(2;5)(p23;q35) t(2;5)(p23;q35) t(2;5)(p23;q35)	*NPM1-ALK* *NPM1-ALK* *NPM1-ALK* *NPM1-ALK*	reference cell line classic/reference cell line
AML myelocytic	EOL-1 HL-60 KASUMI-1 KG-1 NB-4 OCI-AML-3 SKNO-1	Eosinophilic AML, adult, at diagnosis AML M2, adult, at diagnosis AML M2, child, at relapse AML, adult, at relapse AML M3, adult, at relapse AML M4, adult, at diagnosis AML M2, young adult, at relapse	t(8;21(q22;q22) t(15;17)(q22;q11) t(8;21)(q22;q22)	*FIP1L1-PDGFRA*, *KMT2A* ptd *MYC* amp, *NRAS* amp *RUNX1-RUNX1T1*, *KIT* mut *FGFR1OP2-FGFR1* *PML-RARA* *DNMT3A* mut, *NPM1* mut *RUNX1-RUNX1T1*	only eosinophilic cell line classic/reference cell line reference cell line classic/reference cell line classic/reference cell line *NPM1-*mutated cell line
AML monocytic	ME-1 MOLM-13 MONOMAC6 MUTZ-3 THP-1 U-937	AML M4eo, adult, at relapse AML M5a, young adult, at relapse AML M5, adult, at relapse AML M4, young adult, at diagnosis AML M5, infant, at relapse “Hist. lymphoma”, adult, terminal	inv(16)(p13q22) ins(11;9(q23;p22p23) t(9;11)(p22;q23) inv(3), t(12;22)(p13;q12) t(9;11)(p22;q23) t(10;11)(p13;q14)	*CBFB-MYH11* *CBL* mut, *FLT3* itd, *KMT2A-MLLT3* *RUNX1-ATP8A2, KMT2A-MLLT3* *GATA2-EVI1* *CSNK2A1-DDX39B*, *KMT2A-MLLT3* *MLLT10-PICALM*	sAML post-MDS reference cell line dendritic differentiation classic/reference cell line classic/reference cell line
AML erythroid	F-36P HEL OCI-M2 TF-1	AML M6, adult, at diagnosis AML M6, adult, at relapse AML M6, adult, n.r. AML M6, adult, at diagnosis		*CDKN2A* del *CDKN2A* del, *JAK2* mut *RUNX1-TSPEAR* *CBFA2T3-ABHD12*	sAML post-MDS reference cell line sAML post-MDS reference cell line
AML megakaryocytic	CMK ELF-153 M-07e MEGAL MKPL-1 UT-7	AML M7, infant, at relapse AML M7, adult, at relapse AML M7, infant, at diagnosis AML M7, child, n.r. AML M7, adult, at diagnosis AML M7, adult, at diagnosis	del(5)(q13q32)	*CDKN2A* mut, *GATA1* mut, *JAK3* mut *ANO7-DHDH*, *CREBBP* mut *SET-NUP214* *RBM6-CSF1R* biallelic *CDKN2A* del	Down syndrome post-myelofibrosis reference cell line reference cell line
CML myeloid blast crisis	EM-2 K-562 KCL-22 KU-812 LAMA-84	CML, child, blast crisis CML, adult, blast crisis CML, adult, blast crisis CML, adult, blast crisis CML, young adult, blast crisis	t(9;22)(q34;q11) no Ph-chromosome t(9;22)(q34;q11) t(9;22)(q34;q11) t(9;22)(q34;q11)	*BCR-ABL1* e14-a2 *BCR-ABL1* e14-a2, *NUP214-XKR3* *BCR-ABL1* e13-a2, *CEBPA/B* mut *BCR-ABL1* e14-a2 *BCR-ABL1* e14-a2	reference cell line classic/reference cell line reference cell line basophilic differentiation reference cell line
CML lymphoid blast crisis	BV-173 CML-T1 NALM-1 TK-6	CML, adult, blast crisis CML, adult, acute phase CML, child, blast crisis CML, adult, blast crisis	t(9;22)(q34;q11) no Ph-chromosome t(9;22)(q34;q11) t(9;22)(q34;q11)	*BCR-ABL1* e13-a2, *CDKN2A/B* del *BCR-ABL1* e13-a2 *BCR-ABL1* e13-a2 *BCR-ABL1* e14-a2, *MAPK1-AIF1L*,	reference cell line classic/reference cell line
Myeloproliferative Neoplasm	MOLM-20 SET-2	CNL, adult, blast crisis Thrombocythemia, adult, leukemia	t(4;11)(q21;q23) no Ph-chromosome	*KMT2A-SEPT11*, *CSF3R* mut *JAK2* mut	only CNL cell line rare MPN cell line

Discoveries of cytogenetic changes and gene mutational analyses have identified a spectrum of specific genetic alterations in the cell lines of the LL-100 panel representing a highly informative resource in these fields. Please note that this table is selective and does not provide a comprehensive chromosomal and mutational characterization and is by no means an exhaustive list. ^1^ The LL entities for which cell lines were selected are based on the two Revised WHO classifications of myeloid and lymphoid neoplasms [31,32] driving categorization of the cell lines (see also Figure 2). ^2^ All cell lines are available from the DSMZ Cell Lines Bank, a non-profit non-commercial government-owned, public cell line repository (www.dsmz.de). ^3^ Definition of age strata: infant, 0–1 year; child, 1–14 years; adolescent, 14–19 years; young adult, 20–29 years; adult, > 30 years. ^4^ Examples of relevant cytogenetic alterations are listed, e.g., balanced canonical translocation (t) resulting in chimeric fusions listed in the column to the right or rearrangements deregulating oncogenes, deletion (del), inversion (inv), Philadelphia (Ph) or ring chromosome (r). ^5^ Examples of interesting molecular genetic abnormalities and coding alterations are listed, e.g., fusion gene (X-X), gene with amplification (amp), copy number alteration (cna), deletion (del), insertion (ins), internal tandem duplication (itd), mutation (mut), partial tandem duplication (ptd), or rearranged gene (r). The designation of genes follows the terminology approved by the HUGO Gene Nomenclature Committee (www.genenames.org). Older designations are not itemized. ^6^ Unique features: classic/historically important cell lines; virus infection (EBV, Epstein–Barr virus; HHV4, human herpesvirus 4; HIV, human immunodeficiency virus). Some authors have recommended certain cell lines as being particularly well suited to be used as “reference cell lines” [11,29,30] or as “positive control cell lines” in diagnostic procedures applying standardized cell line-based DNA controls [35,36,37,38,39]. Abbreviations of disease entities: ABC, activated B-cell; AIDS, acquired immunodeficiency syndrome; ALCL, anaplastic large cell lymphoma; ALL, acute lymphoblastic leukemia; AML, acute myeloid leukemia; BCP, B-cell precursor; CLL, chronic lymphocytic leukemia; CML, chronic myeloid leukemia; CNL, chronic neutrophilic leukemia; CTCL, cutaneous T-cell lymphoma; DLBCL, diffuse large B-cell lymphoma; GCB, germinal center B-cell; HCL, hairy cell leukemia; LGL, large granular lymphocyte (leukemia); Mal., malignant; MCL, mantle cell lymphoma; MDS, myelodysplastic syndromes; MPN, myeloproliferative neoplasm; NHL, non-Hodgkin lymphoma; NK, natural killer; n.r., not reported; PCL, plasma cell leukemia; PEL, primary effusion lymphoma; PLL, prolymphocytic leukemia; PMBL, primary mediastinal B-cell lymphoma; sAML, secondary AML.

**Table 3:** Unique Features and Benefits of the LL-100 Panel.

Criterion	Implementation
Authentication	All cell lines are continuously and unequivocally authenticated and validated
Derivation	Cell lines are assigned to the verified tissue
Microbial/viral contamination	Cell lines are free of mycoplasma and non-inherent viruses
Long-term culture	Passage numbers are kept low, no extended cultivation, frozen storage in liquid nitrogen
Methodology	Methods of RNA and DNA isolation and sequencing were identical for all cell lines
Data availability	Whole exome and RNA sequence data are freely available
Cell line availability	All cell lines are publicly available
